# Infección por SARS-CoV-2 y su impacto en la enfermedad hepática

**DOI:** 10.1515/almed-2022-0010

**Published:** 2022-06-01

**Authors:** Sergio Salgüero Fernández, Pablo Gabriel Medina, Alejandro Almería Lafuente, María Antonieta Ballesteros Vizoso, Angielys Zamora Trillo, Gregori Casals Mercadal, Gemma Solé Enrech, Marta Lalana Garcés, Armando R. Guerra Ruiz, Oihana Ortiz Pastor, Manuel Morales Ruiz

**Affiliations:** Comisión de valoración bioquímica de la enfermedad hepática, Sociedad Española de Medicina de Laboratorio (SEQC-ML), Barcelona, Spain; Servicio de Análisis Clínicos, Hospital Universitario Fundación Alcorcón, Madrid, Zaragoza, España; Servicio de Bioquímica, Hospital Universitari Vall d’Hebron, Barcelona, Zaragoza, España; Servicio de Bioquímica Clínica, Hospital Royo Villanova, Zaragoza, España; Servicio de Análisis Clínicos, Hospital Universitario Son Espases. Palma de Mallorca, Zaragoza, España; Servicio de Bioquímica Clínica, Hospital General Universitario Gregorio Marañón, Madrid, Spain; Servicio de Bioquímica y Genética Molecular, CDB, Hospital Clínic de Barcelona, IDIBAPS, CIBERehd, Barcelona, Spain; Servicio de Laboratorio, UDIAT-CD. Corporació Sanitaria Parc Taulí, Sabadell, España; Servicio de Análisis Clínicos, Hospital de Barbastro, Huesca, España; Servicio de Análisis Clínicos, Hospital Universitario Marqués de Valdecilla, Santander, España; Servicio de Bioquímica, Hospital Universitario Miguel Servet, Zaragoza, España; Servicio de Bioquímica y Genética Molecular, CDB, Hospital Clínic de Barcelona, IDIBAPS, CIBERehd, Departamento de Biomedicina de la Facultad de Medicina y Ciencias de la Salud-Universidad de Barcelona, Barcelona, España

**Keywords:** daño hepático, transaminasas, virus

## Abstract

**Introducción:**

En el contexto de la infección por SARS-CoV-2 no es infrecuente encontrar alteraciones hepáticas, tanto en pacientes con enfermedad hepática crónica previa como sin ella.

**Contenido:**

En esta revisión, se examina el conocimiento actual sobre la relación entre la COVID-19 y el daño hepático, frecuentemente observado en el seno de esta enfermedad.

**Resumen:**

Si bien no está completamente dilucidada la patogénesis del daño hepático, parece ser consecuencia de la combinación de varios factores, entre los que se encuentran el daño directo del virus, el derivado de la hiperactivación del sistema inmune, el isquémico y el farmacológico. El valor pronóstico de estas alteraciones también está bajo intensa investigación. La potencial repercusión de las mismas aboga por la necesidad de adecuar el manejo y el tratamiento de los pacientes, particularmente en el contexto de pacientes con enfermedad hepática crónica o trasplantados hepáticos.

**Perspectiva:**

Se desconocen actualmente muchos aspectos relativos a la afectación hepática durante la COVID-19, particularmente en las formas graves de la enfermedad. El desarrollo de nuevos estudios referidos a las implicaciones clínicas de la COVID-19 en el hígado, tanto en estado sano como enfermo, podrían ayudar a ajustar las recomendaciones de tratamiento y vacunación según el perfil del paciente.

## Introducción

La enfermedad causada por SARS-CoV-2, denominada como COVID-19, se ha extendido rápidamente de forma global ocasionando una pandemia con repercusiones clínicas, humanitarias y económicas sin precedentes en la edad moderna [1, 2]. El número de casos y fallecidos a la fecha actual puede monitorizarse desde una plataforma creada por la Universidad John Hopkins (https://coronavirus.jhu.edu/map.html). Los datos acumulados hasta el momento en la Unión Europea muestran que, entre los casos confirmados, el 30% requirieron ingreso y el 4% necesitaron asistencia en las unidades de cuidados intensivos (UCI) debido a su estado crítico [3]. La mortalidad es particularmente elevada en pacientes con edad avanzada y comorbilidades preexistentes, como obesidad, enfermedades cardiovasculares y diabetes [4]. Estos datos evolucionan a la vez que la pandemia y la aparición de nuevas variantes con una menor patogenicidad [5].

La causa de la muerte suele ser insuficiencia respiratoria aguda secundaria a daño alveolar difuso, pero el hígado puede ser un órgano clave durante el desarrollo de la infección [6].

Por un lado, la infección por SARS-CoV-2 repercute en la elevación de marcadores de lesión hepática en pacientes sin historia previa de disfunción hepática. Estos marcadores tienden a elevarse coincidiendo con la duración de hospitalización y el estado de gravedad. En este contexto, diversos estudios han mostrado elevación de alanina aminotransferasa (ALT), aspartato aminotransferasa (AST), bilirrubina total y gamma-glutamil transferasa (GGT) por encima de 3 veces el límite superior de normalidad (LSN) [7, 8]. El uso de medicamentos específicos para el tratamiento contribuye en parte a la elevación de estos marcadores, lo que justifica el seguimiento de los parámetros hepáticos en estos pacientes.

Por otro lado, las evidencias recientes también muestran que la enfermedad hepática crónica es otro factor de riesgo que impacta en los pacientes con COVID-19, como muestran los registros internacionales multicéntricos COVID-Hep.net y COVIDCirrhosis.org [9]. Estos datos indican que los pacientes con cirrosis tienen un mayor riesgo de descompensación y mortalidad asociada a la COVID-19, especialmente entre aquellos con cirrosis más avanzada y con enfermedad hepática relacionada con el alcohol [9]. El tropismo del SARS-CoV-2 hacia los colangiocitos [10], la disfunción inmunológica asociada a la enfermedad hepática crónica [11], así como otros factores de riesgo en estos pacientes, pueden explicar la alta incidencia de mortalidad debida a complicaciones relacionadas con la función hepática.

En vista de las evidencias anteriores que relacionan el impacto de la COVID-19 sobre el hígado, las principales sociedades científicas en el campo de la hepatología (*American Association for the Study of Liver Diseases* o AASLD, European Association for the Study of the Liver o EASL y la *Asian Pacific Association for the Study of the Liver* o APASL), han elaborado documentos de posicionamiento que recomiendan marcos generales de actuación [12], [13], [14].

En esta línea, esta revisión discute la patogénesis de la infección por SARS-CoV-2, su impacto en la disfunción hepática y necesidades de seguimiento. Todo ello contextualizado en pacientes con enfermedad hepática previa.

## Alteración de los marcadores bioquímicos hepáticos

La alteración de los marcadores bioquímicos hepáticos es común durante la infección por SARS-CoV-2, oscilando su ocurrencia entre un 15–65% de los pacientes [15]. Estudios previos ya habían mostrado que el daño hepático era un hecho común en pacientes infectados con otros dos coronavirus altamente patogénicos como son el SARS-CoV y el MERS-CoV [16].

La alteración más frecuente observada es la elevación leve, 1–2 veces el LSN, de las enzimas de citólisis, AST (38–63% de los pacientes) y ALT (29–39%). Aunque infrecuentes, se han descrito elevaciones de transaminasas superior a 5 veces el LSN, llegándose a originar una hepatitis aguda grave en algunos casos [17].

La AST suele estar más elevada que la ALT lo que podría reflejar una lesión no hepática (por ejemplo, miositis) [17]. Sin embargo, los niveles de AST correlacionan positivamente con los de ALT, pero no con los de rabdomiólisis, como los de creatina quinasa, o los de inflamación sistémica, como los de ferritina o proteína C reactiva (PCR), lo que sugiere daño hepático [15]. Aunque se desconocen los mecanismos de esta elevación preferencial de la AST, pueden estar involucrados aspectos como la disfunción mitocondrial, la esteatosis y la alteración de la perfusión hepática [15].

Por el contrario, es más rara la elevación de fosfatasa alcalina (6% de los pacientes) y GGT (21%) y la bilirrubina total suele permanecer normal o discretamente elevada [15, 17].

El valor pronóstico de estas alteraciones no está por ahora dilucidado. Por un lado, se ha observado que la presencia de daño hepático es más frecuente en los casos graves de COVID-19 que en los leves [15, 17, 18]. Varios estudios correlacionan las elevaciones de los marcadores hepáticos con mal pronóstico, incluyendo admisión a la UCI, necesidad de ventilación mecánica o mortalidad [15, 17, 18]. Además, la hipoalbuminemia también se ha asociado a un peor pronóstico como marcador inespecífico de gravedad [15, 17].

Sin embargo, otros estudios no han podido detectar la misma correlación pronóstica y apuntan hacia el hecho que, si bien la alteración bioquímica es común, puede no tener grandes consecuencias clínicas y apartar el foco de lo realmente importante que es controlar la disfunción inmune generada en el seno de la enfermedad [19, 20].

**Figura 1:**
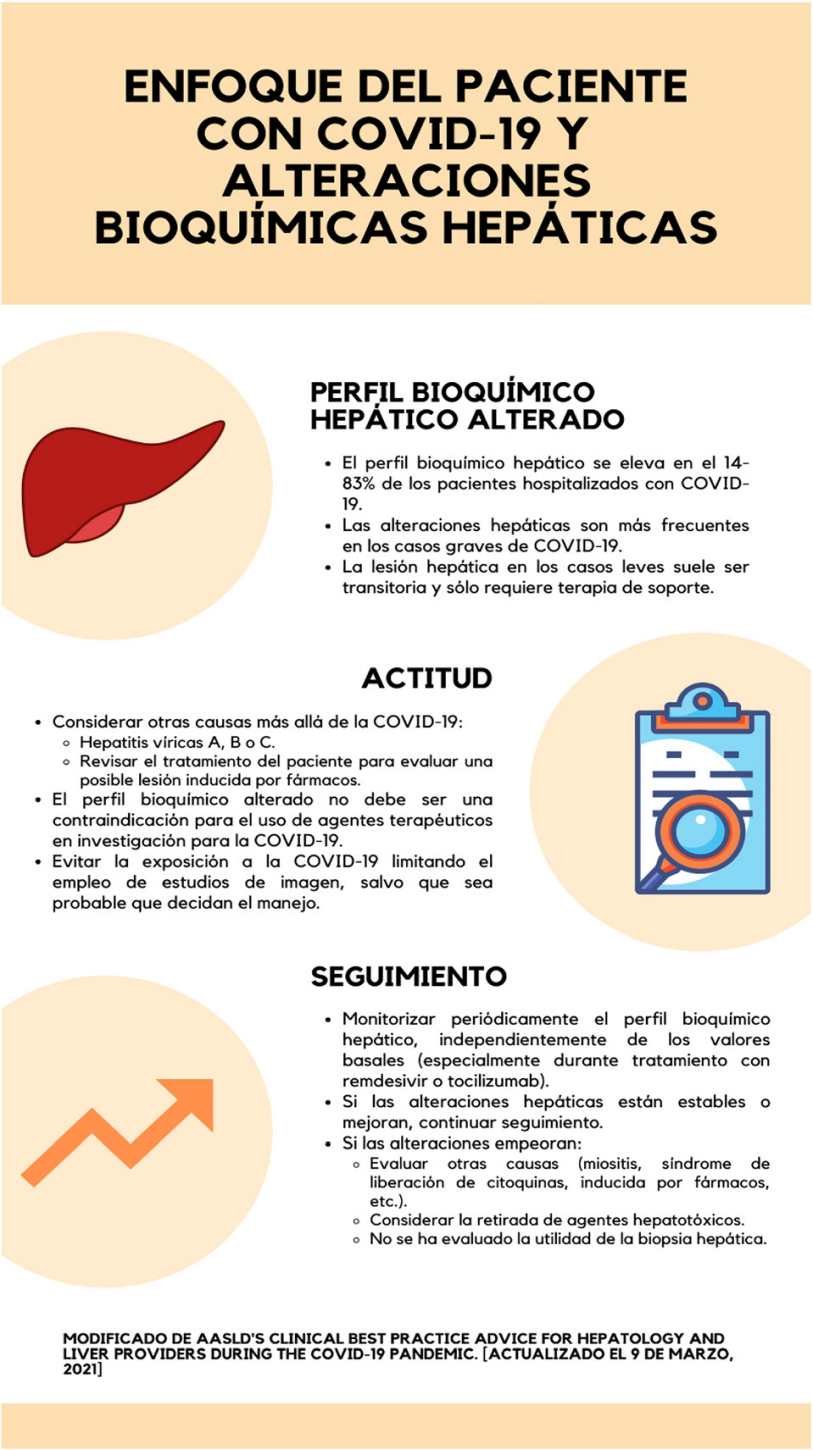
Recomendaciones para el manejo del paciente con COVID-19 y descripción de las alteraciones bioquímicas hepáticas (modificado a partir de [15]).

El daño hepático en los casos leves de COVID-19 suele ser transitorio y solo requiere tratamiento de soporte, sin necesitar tratamiento específico. Por otro lado, es difícil distinguir si las alteraciones de los marcadores bioquímicos hepáticos se deben a la infección viral propiamente o a sus complicaciones (miositis, síndrome de liberación de citoquinas, isquemia/hipotensión) o al tratamiento empleado. Debido a lo previo y la existencia de controversia sobre la relevancia de las alteraciones de los marcadores hepáticos, la declaración de consenso del panel de expertos de la AASLD opta por un manejo clínico expectante intentando descartar otras etiologías y/o fuentes de daño hepático (Figura 1). En cualquier caso, el manejo de cada paciente debe ser flexible y personalizado, particularmente en aquellos con una enfermedad hepática crónica subyacente [21], como se describirá posteriormente.

Finalmente, la vacunación contra la COVID-19 se ha relacionado con la aparición de dos síndromes con manifestaciones hepáticas como la trombocitopenia trombótica y daño hepático agudo, similar al autoinmune. La trombocitopenia trombótica se ha relacionado con el uso de vacunas basadas en adenovirus y parece estar mediada por el desarrollo de anticuerpos que reconocen el factor plaquetario 4 [22, 23]. La aparición de daño hepático similar al autoinmune se describió primero con las vacunas de ARN mensajero, aunque ya se ha reportado con ambos tipos de vacunas [23], [24], [25]. Es un diagnóstico de exclusión y es difícil demostrar un efecto causal de la vacunación en la aparición de la hepatitis autoinmune ya que puede representar una aparición espontánea en personas susceptibles, coincidente en el tiempo [23], [24], [25]. Ambos síndromes son complicaciones raras, con una frecuencia estimada en 1 de cada millón de vacunas, y no afectan las recomendaciones generales sobre la vacunación [23].

## Patogénesis del daño hepático

SARS-CoV-2 es capaz de penetrar la célula diana gracias a la unión de la proteína de la espícula (S), con el receptor 2 (ACE2) de la enzima convertidora de la angiotensina (ECA) [15]. La proteína S requiere ser activada por la serina proteasa TMPRSS2 de la célula hospedadora para su entrada en la célula [26]. Es significativa la asociación de distintas variaciones individuales de esta proteasa con el pronóstico de la enfermedad y cuya inhibición es un objetivo terapéutico potencial [27].

Se ha demostrado la expresión de ACE2 a nivel hepático en las células epiteliales de los conductos biliares llamadas colangiocitos (comparable a la de neumocitos tipo 2 del pulmón, 60%), células endoteliales sinusoidales y hepatocitos (3%) y ésta podría verse incrementada con el daño hepático e inflamación [28]. La alta expresión en colangiocitos, significativamente mayor que en la población de hepatocitos (59, 7% vs. 2, 6%) [28], [29], [30], contrasta con la ausencia de un patrón bioquímico colestásico, como se ha mencionado previamente.

**Figura 2:**
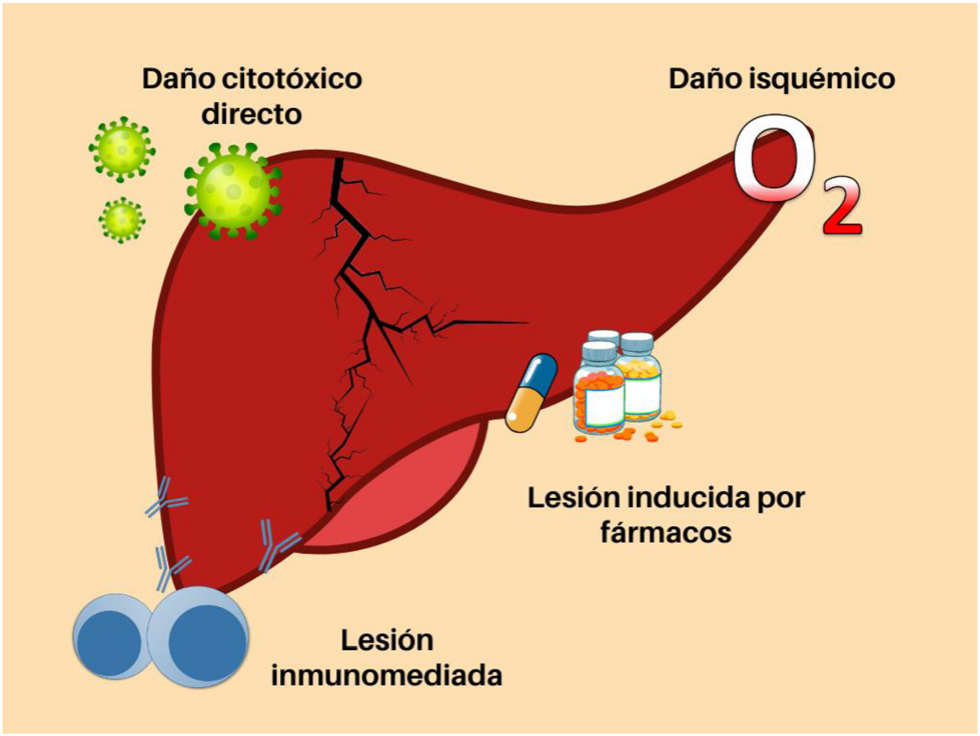
Mecanismos generadores de daño hepático en pacientes infectados con SARS-CoV-2.

La lesión hepática asociada a la COVID-19 se define como cualquier daño hepático que se produzca durante el curso de la enfermedad y el tratamiento de los pacientes [28]. En la mayoría de los casos, la patogénesis parece ser multifactorial (Figura 2), coexistiendo varios mecanismos como la citotoxicidad directa a debido a la replicación del virus en el hígado, la hiperactivación inmune, el daño isquémico y la toxicidad farmacológica [29].

El daño hepatocelular asociado a COVID-19 se caracteriza principalmente por la presencia de esteatosis moderada, inflamación lobular y portal, focos apoptóticos/necróticos y elevación de ALT y AST en plasma. Esta lesión podría estar causada por replicación directa del virus en el hígado que induce una disfunción mitocondrial y estrés del retículo endoplásmico, contribuyendo a la esteatosis, y produciendo daño hepático [28].

SARS-CoV-2 codifica diversas proteínas estructurales y no estructurales (NSP) que suprimen la respuesta antiviral, fundamentalmente mediada por interferón, mediante una multitud de estrategias [31]. Por ejemplo, NSP1 actúa inhibiendo la traducción en la célula huésped [32]. Además, la infección por SARS-CoV-2 podría también activar mTOR, lo que finalmente inhibe la autofagia (como mecanismo de degradación viral) y facilita el escape viral del sistema inmunitario [28]. La acción directa de algunas de estas proteínas virales sobre la función hepática no se ha demostrado, pero es concebible dada la capacidad viral para alterar la maquinaria proteica de la célula huésped [31, 33]. El efecto citopático viral directo sobre las células que expresan ACE2 y TMPRSS2 en el hígado podría estar detrás de la mayor parte de la lesión hepática causada de forma directa. En este sentido, los colangiocitos, con funciones fisiológicas en la respuesta inmune adaptativa y regeneración del hígado, se han propuesto como los principales afectados [10, 34].

Otros autores han desestimado la acción directa del virus sobre el hígado argumentando que la asociación pronóstica de los marcadores hepáticos es un reflejo de la desregulación inmune existente, que afecta la interacción de las células T citotóxicas intrahepáticas con las células de Kupffer, como se ha reportado en el contexto de otras infecciones virales respiratorias, y en ausencia de replicación intrahepática [20].

Los datos histopatológicos de los que se disponen no ayudan a esclarecer esta situación. En algunos estudios los patrones histopatológicos analizados, así como la presencia de ARN viral, parecen ser consistentes con daño hepático mediado por el virus [35], [36], [37]. Entre los principales cambios post-mortem observados se encuentran la esteatosis (predominantemente macrovesicular), la hepatitis aguda leve (afectación lobular necroinflamatoria) y la inflamación portal leve [29, 36]. Sin embargo, otros autores apoyan la hipótesis que el hígado no debe ser la principal fuente de preocupación y que el nivel elevado de transaminasas detectado en algunos pacientes parece estar más relacionado con la disfunción endotelial y coagulopatía característica de estos pacientes [38, 39].

Estos datos, más que ser contradictorios, probablemente sean un reflejo del origen multifactorial del daño hepático producido durante la COVID-19. Por tanto, el patrón de lesión histológica no es específico de una etiología.

La infección por SARS-CoV-2 se asocia a inflamación sistémica caracterizada por altos niveles de citoquinas proinflamatorias que podrían estar detrás de los elevados niveles de transaminasas observados. Así, se ha observado como los niveles de ALT parecen correlacionar con los de PCR, dímero-D, ferritina o IL-6 [15]. Una hiperactivación proinflamatoria excesiva del sistema inmunitario, la llamada “tormenta” de citoquinas, puede ser más dañina que el efecto citopático del virus *per se*. En este sentido, los niveles de IL-6 correlacionan con la evolución de la enfermedad y su inhibición (ej. mediante tocilizumab) es una vía de terapia frente a esta grave complicación [6, 15]. Por otro lado, la disfunción del sistema inmune es una característica crucial durante el proceso fibrótico [40], por lo que la inflamación causada por el virus podría contribuir a la aparición de fibrosis, la cual ha sido observada en pacientes con COVID-19 [41].

El daño isquémico puede tener su papel en la lesión hepática. Los eventos trombóticos ocurren con frecuencia en el seno da la enfermedad COVID-19 y se asocian a un peor pronóstico. El estado proinflamatorio, antes mencionado, puede favorecer la formación de trombos inmunológicamente mediados que afecten fundamentalmente a la microcirculación [38]. Se ha detectado una prevalencia del 29% de trombosis microvascular intrahepática en pacientes con COVID-19 lo que podría causar hipoperfusión hepática. Otros contribuyentes podrían ser la congestión hepática debido a cardiomiopatía o la hipoxia sistémica ya observada en el contexto de otras neumonías virales [6, 15].

Además, los fármacos utilizados en el curso de la enfermedad pueden contribuir al daño hepático, describiéndose casos asociados al uso de remdesivir, lopinavir o tocilizumab [6, 15]. De especial interés, son aquellos pacientes con una enfermedad hepática crónica preexistente que pueden presentar un riesgo de toxicidad mayor o pacientes trasplantados donde se pueden dar interacciones indeseadas con los fármacos inmunosupresores [6, 15].

## Evolución de la COVID-19 en pacientes con enfermedad hepática crónica

La prevalencia de enfermedad hepática crónica previa en series extensas de pacientes hospitalizados con enfermedad COVID-19 severa se ha estimado entre un 0, 6 y un 1,4% [42], [43], [44]. Aunque la naturaleza de la hepatopatía es determinante a la hora de evaluar el pronóstico de la COVID-19, se ha reportado que aproximadamente un 60% de los pacientes con enfermedades hepáticas crónicas tenían un cuadro de infección grave y con una tasas de mortalidad del 18% [45].

Las alteraciones metabólicas presentes en pacientes con obesidad o con enfermedad del hígado graso asociada a la disfunción metabólica (MAFLD, por sus siglas en inglés), junto con otros factores de riesgo, como edad y sexo masculino, se han asociado a un peor pronóstico de la enfermedad [46], [47], [48]. Además, los marcadores bioquímicos hepáticos de estos pacientes con disfunción metabólica se encuentran más alterados durante el ingreso hospitalario [47, 49]. No obstante, el perfil de estos pacientes suele incluir la presencia de hipertensión arterial, diabetes, enfermedad cardiovascular o enfermedad pulmonar obstructiva crónica, por lo que el papel de la hepatopatía crónica en la gravedad de la COVID-19 ha de ser analizado con detenimiento [50].

La cirrosis hepática la podemos considerar como uno de los factores de peor pronóstico para la COVID-19, hecho común a otras infecciones. A medida que el grado de cirrosis aumenta, como reflejan los distintos estadios de la clasificación Child-Pugh, se incrementa la mortalidad y el requerimiento de ingreso a UCI [9, 51]. Además, la propia infección por SARS-CoV-2 actúa como desencadenante de descompensación hepática, favoreciendo el aumento de inflamación sistémica, desarrollo de encefalopatía, desregulación del sistema inmune y coagulopatía. La combinación de los registros internacionales SECURE-Cirrhosis y COVID-HEP recopila una serie de datos actualizados que incluyen múltiples pacientes con cirrosis y COVID-19 (https://www.covid-hep.net/updates.html) y en el que la mortalidad en estos pacientes es del 38% (unas 5 veces mayor que la observada en la población general de la misma edad) llegando a ser cercana al 70% en los pacientes con estadio Child-Pugh C [9]. Aunque la principal causa de muerte fue el fallo respiratorio, la descompensación hepática aguda apareció en casi la mitad de los pacientes con cirrosis, de los cuales el 21% no presentaba síntomas respiratorios [9].

Las hepatopatías crónicas de origen infeccioso por virus hepatotropos, como es el caso del virus de la hepatitis B, no parecen predisponer a un peor pronóstico de COVID-19 [52]. Sin embargo, el tratamiento inmunosupresor empleado para combatir la “tormenta” de citoquinas (glucocorticoides, tocilizumab, etc.) puede incrementar el riesgo de reactivación viral [6]. Se ha detectado un aumento reversible de las transaminasas con el uso de estos fármacos [53], siendo desaconsejables si los valores de AST y/o de ALT superan 5 veces o más el LSN [54].

En el caso de las hepatopatías de origen autoinmune, no ocasionan un aumento de la mortalidad con respecto a la población general. El hecho de que se encuentre tratada con fármacos inmunomoduladores o inmunosupresores no supone un mayor riesgo de complicaciones asociadas a la COVID-19, por lo que se recomienda no reducir el tratamiento inmunosupresor durante el curso de la enfermedad [15].

En cuanto a los pacientes con carcinoma hepatocelular, no existe demasiada información sobre el pronóstico que ocasiona en la COVID-19. Los tratamientos inmunosupresores y con agentes quimioterapéuticos pueden favorecer la infección por SARS-CoV-2 y el desarrollo de enfermedad severa, sugiriendo un peor pronóstico [55].

Respecto a los trasplantes hepáticos, éstos han disminuido drásticamente durante la pandemia, debido a la elevada morbilidad y mortalidad de los pacientes trasplantados contagiados por SARS-CoV-2, la necesidad de reservar las UCI a los pacientes con COVID-19 grave y el aumento del riesgo de infección viral tras el inicio del tratamiento inmunosupresor [50]. La disminución del número de donantes y el riesgo a una infección nosocomial han limitado los trasplantes a aquellos casos más severos, con riesgo de fallo hepático fulminante [6]. Por otro lado, los pacientes receptores del trasplante son en mayor medida hombres, con una edad media de 56 años y alta prevalencia de diabetes y obesidad, factores asociados a una COVID-19 severa. Aún hay aspectos desconocidos sobre la susceptibilidad de los trasplantados hepáticos al SARS-CoV-2, como un mayor riesgo de reinfección debido a una peor respuesta inmune adaptativa, la duración de IgG neutralizantes, el periodo de diseminación viral o el desarrollo de otros síntomas poco comunes [15].

## Manejo de los pacientes con COVID-19 y enfermedad hepática crónica

Existen una serie de recomendaciones en función de la etiología de la hepatopatía en relación al manejo y tratamiento de las personas con enfermedad hepática crónica. En general, se recomienda reducir al máximo las interacciones sociales y el uso de telemedicina para los controles rutinarios [50].

La MAFLD requiere una intervención más intensa en el estilo de vida, incluyendo pérdida de peso y control de la diabetes, con el fin de evitar desbalances metabólicos que puedan agravar un potencial contagio por SARS-CoV-2. Se ha de continuar el tratamiento de la hipertensión, aunque sean inhibidores de la ECA. Se recomienda ingreso hospitalario temprano en caso de infección por SARS-CoV-2 [21].

Los enfermos de hepatitis B o C crónica han de continuar su tratamiento antiviral ya que no lleva asociado una mayor probabilidad de contagio por SARS-CoV-2. Se aconseja establecer el estado serológico previo al empleo de fármacos inmunosupresores potentes debido al riesgo de reactivación viral, así como evaluar la necesidad de tratamiento antiviral profiláctico [6, 21].

Los enfermos con cirrosis hepática son vulnerables al desarrollo de COVID-19 severa, por lo que, ante descompensaciones hepáticas, se requiere el testeo para SARS-CoV-2 independientemente de la sintomatología respiratoria. En caso de infección, aun con clínica leve, se recomienda el ingreso hospitalario e iniciar tratamiento antiviral lo antes posible [15, 56]. Además, se recomienda su vacunación frente a virus de la gripe y *Streptococcus pneumoniae* [21].

Por otro lado, la cirrosis y el estado de disfunción inmune que acarrea ya se ha relacionado con baja respuesta al empleo de otras vacunas, por lo que se necesitan más estudios que evalúen las distintas vacunas frente a SARS-CoV-2 y la posible necesidad de dosis y/o intervalos ajustados en esta población [57].

Las personas afectas de hepatocarcinoma y las que poseen un mayor riesgo de su desarrollo (cirrosis, hepatitis crónica, etc.) deben seguir bajo vigilancia, ya que se consideran personas con peor pronóstico a la COVID-19 [21]. No obstante, se ha reportado una disminución en los diagnósticos de hepatocarcinoma durante la pandemia [15].

En cuanto a los pacientes trasplantados hepáticos, se recomienda continuar el tratamiento inmunosupresor, y únicamente disminuirlo para reducir el riesgo de linfopenia o infecciones oportunistas en caso de contagio por SARS-CoV-2, sin aumentar el riesgo de rechazo del injerto. Se han de tener en cuenta las interacciones entre los inhibidores de la calcineurina y la terapia anti SARS-CoV-2, rechazando la coadministración de anticuerpos monoclonales o monitorizando la concentración del fármaco inmunosupresor [21, 29]. Los trasplantados hepáticos tienen reducida la tasa de generación anticuerpos en respuesta a la vacunación frente al SARS-CoV-2 [15].

En cuanto al uso de las vacunas en pacientes con enfermedad hepática crónica, cáncer hepatobiliar o en pacientes receptores de trasplante hepático, la EASL elaboró una serie de recomendaciones, que incluyen la vacunación de estos pacientes, así como la priorización de los mismos y de sus cuidadores (familiares, sanitarios) en el proceso. Asimismo, se deben establecer registros prospectivos que permitan monitorizar la seguridad, inmunogenicidad y efectividad de las diferentes vacunas empleadas en pacientes con enfermedades hepáticas crónicas y receptores de trasplantes hepáticos [58].

## Conclusiones

El efecto de la COVID-19 sobre el hígado no está claro. Cada vez más estudios parecen apoyar una causa multifactorial de la infección viral en el daño hepático. Si bien las alteraciones hepáticas son frecuentes durante la enfermedad, se desconoce si son causa o consecuencia de una peor evolución de la COVID-19. Sin embargo, el obviar las posibles repercusiones de la infección viral en el hígado, especialmente en pacientes con daño hepático previo o inmunosuprimidos, puede tener consecuencias fatales para estos pacientes. Se requieren más estudios que evalúen el valor pronóstico de las alteraciones hepáticas, tan frecuentemente observadas, y la posibilidad de establecer un manejo y/o tratamiento específico de las mismas. Asimismo, los pacientes con enfermedad hepática previa se podrían beneficiar de un análisis más exhaustivo de la respuesta a la vacunación en este grupo de pacientes, particularmente con la aparición de nuevas variantes. En este sentido, la creación de registros multicéntricos internacionales puede facilitar esta tarea, aunque deberá ser complementada con estudios moleculares y traslacionales.
